# The Health Economic Value of Changes in Glycaemic Control, Weight and Rates of Hypoglycaemia in Type 1 Diabetes Mellitus

**DOI:** 10.1371/journal.pone.0162441

**Published:** 2016-09-15

**Authors:** Phil McEwan, Hayley Bennett, Jonathan Fellows, Jennifer Priaulx, Klas Bergenheim

**Affiliations:** 1 Health Economics and Outcomes Research Ltd., Mulberry Drive, Cardiff, United Kingdom; 2 Global Health Economics and Outcomes Research, AstraZeneca, London, United Kingdom; 3 Global Health Economics and Outcomes Research, AstraZeneca, Molndal, Sweden; Baylor College of Medicine, UNITED STATES

## Abstract

**Aims:**

Therapy-related consequences of treatment for type 1 diabetes mellitus (T1DM), such as weight gain and hypoglycaemia, act as a barrier to attaining optimal glycaemic control, indirectly influencing the incidence of vascular complications and associated morbidity and mortality. This study quantifies the individual and combined contribution of changes in hypoglycaemia frequency, weight and HbA1c to predicted quality-adjusted life-years (QALYs) within a T1DM population.

**Materials and methods:**

We describe the Cardiff Type 1 Diabetes (CT1DM) Model, originally informed by the Diabetes Control and Complications Trial (DCCT) and updated with the Epidemiology of Diabetes Interventions and Complications (EDIC) study and Swedish National Diabetes Registry for microvascular and cardiovascular complications respectively. We report model validation results and the QALY impact of HbA1c, weight and hypoglycaemia changes.

**Results:**

Validation results demonstrated coefficients of determination for clinical endpoints of R^2^ = 0.863 (internal R^2^ = 0.999; external R^2^ = 0.823), costs R^2^ = 0.980 and QALYs R^2^ = 0.951. Achieving and maintaining a 1% HbA1c reduction was estimated to provide 0.61 additional discounted QALYs. Weight changes of ±1kg, ±2kg or ±3kg led to discounted QALY changes of ±0.03, ±0.07 and ±0.10 respectively, while modifying hypoglycaemia frequency by -10%, -20% or -30% resulted in changes of -0.05, -0.11 and -0.17. The differences in discounted costs, life-years and QALYs associated with HbA1c 6% versus 10% were -£19,037, 2.49 and 2.35 respectively.

**Conclusions:**

Using a model updated with contemporary epidemiological data, this study presents an outcome-focused perspective to assessing the health economic consequences of differing levels of glycaemic control in T1DM with and without weight and hypoglycaemia effects.

## Introduction

Type 1 diabetes mellitus (T1DM) is a chronic autoimmune disorder associated with significant excess morbidity and mortality [1, 2]. It is estimated that 8.5% of those diagnosed with diabetes in the UK have T1DM; equating to 284,405 people and representing a 0.4% prevalence rate [3].

Treatment of T1DM typically requires multiple daily injections of insulin with therapeutic guidelines advocating the use of patient optimised management strategies and individualised targets [4]. However, despite such guidelines, fewer than 30% of UK T1DM adults reach treatment targets for glucose control, with the disease reducing adult life expectancy in the UK by approximately 13 years [5].

Therapy-related consequences of treatment, such as weight gain and hypoglycaemia are known to act as a potential barrier to attaining optimal glycaemic control [6] and may therefore indirectly influence the incidence of vascular complications. Furthermore, the independent impact of hypoglycaemia and weight gain upon quality of life has been well documented [7–9]. Consequently, changes in HbA1c, weight and the frequency of hypoglycaemia are important, inter-related determinants of the cost effectiveness of therapeutic interventions. This is of particular relevance to the management of T1DM as the risk of recurrent hypoglycaemia in insulin treated patients is high [10] and the prevalence of obesity amongst T1DM patients has increased significantly over recent years [11].

Previous health economic analysis has characterised the relative impact of weight change, hypoglycaemia frequency and unit changes in HbA1c upon predicted quality-adjusted life years (QALYs) in type 2 diabetes mellitus [12]; however, such analyses have not been undertaken in T1DM. Consequently, the principle objective of this study was to quantify the individual and combined contribution of changes in hypoglycaemia frequency, weight and HbA1c to predicted quality-adjusted life years (QALYs) in a T1DM population. A secondary objective was to quantify the health economic value associated with improvements in glycaemic control in T1DM.

## Materials and Methods

**Fig 1:**
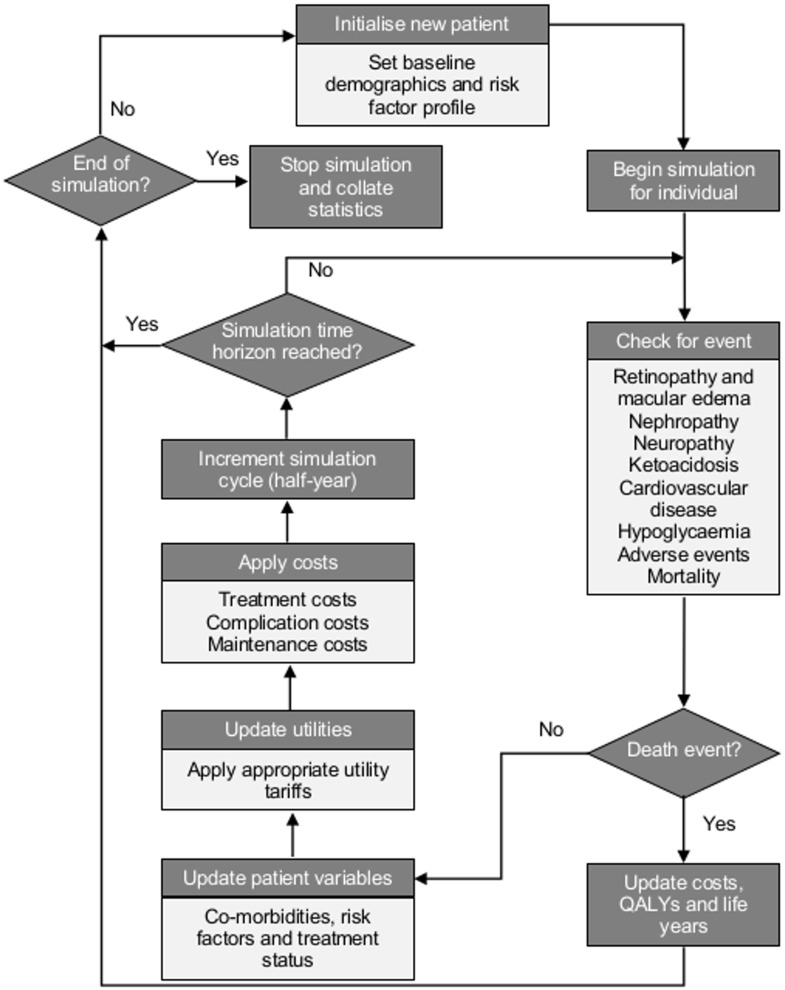
Flow diagram of the Cardiff Type 1 Diabetes Model simulation process.

The Cardiff Type 1 Diabetes (CT1DM) Model is a fixed-time-increment stochastic simulation model designed to evaluate the lifetime impact of therapeutic changes on individual simulated patients. The model was originally designed in 2009 and based on the original CORE T1DM model [13] with disease progression data being predominantly drawn from the Diabetes Control and Complications Trial (DCCT) [1] for microvascular complications and Framingham [14] for cardiovascular complications. Consistent with both established and recently published T1DM models [15]] the model has been updated to include long-term epidemiological evidence from the DCCT follow-up study—the Epidemiology of Diabetes Interventions and Complications (EDIC) [2] study and also the T1DM specific Swedish National Diabetes Registry [16] cardiovascular risk equations. Fig 1 shows the model’s flow diagram.

### Microvascular event rates

The estimation of transition probabilities for microvascular health states was undertaken using a similar approach to that reported by Lung et al. [17] in which time-dependent parametric Weibull regression equations were fitted to cumulative incidence data from DCCT and EDIC.

Specifically, regression models were fitted to cumulative incidence of retinopathy and macular edema using EDIC [18] with simulated patients capable of progressing from no retinopathy to background diabetic retinopathy (BDR), to proliferative diabetic retinopathy (PDR) and to severe vision loss (SVL) with macular edema a separate health state associated with increased risk of SVL.

Patients can progress from no nephropathy to micro-albuminuria [19], from which they may either return to no nephropathy or progress to macro-albuminuria with or without impaired glomerular filtration rate (GFR) and finally to end stage renal disease (ESRD) [20]. Upon progression to ESRD patients can receive transplant [21], experience graft failure and return to dialysis [22], or die either whilst receiving dialysis [23] or from the functioning graft health state [22].

Patients may progress from no neuropathy to diabetic peripheral neuropathy [24] with rates controlling progression to foot ulcers, deep foot infections, amputations and event specific mortality taken from a previously published Markov model [25]. Specific details of the rates and Weibull regression models used to control simulated patients thought the various microvascular health states together with the risk factor variables that influence the likelihood of progression are detailed in Appendix 1.

### Cardiovascular disease

Modelling cardiovascular disease (CVD) was implemented using an equation derived from the Swedish National Diabetes Registry in which 3,661 subjects with T1DM were followed up for five years [16]. Risk of CVD (defined as fatal or non-fatal myocardial infarction or stroke events) from this study was significantly associated with age, duration of T1DM, total cholesterol to high-density lipoprotein (HDL) cholesterol ratio, systolic blood pressure (SBP) and the following binary variables: current smoker, presence of macroalbuminuria and a history of prior CVD. The Swedish Equation for CVD does not partition events into fatal or non-fatal; consequently we assume a fixed proportion (39.19%) are fatal [26].

### Hypoglycaemia

Hypoglycaemia is modelled utilising therapy-related event rates, categorised as a daytime or nocturnal non-severe hypoglycaemic event (NSHE) or as a severe hypoglycaemic event (SHE). The occurrence of an event can be associated with a cost [27] and a decrease in quality of life [7].

### Ketoacidosis

Ketoacidosis is modelled as an acute event health state. Patients may have a ketoacidosis event during any cycle of the modelled time horizon and may have multiple events over a lifetime. The rate of ketoacidosis incidence applied in the model is 1.585 per 100,000 persons and is taken from the Swedish study by Wang et al. [28].

### Baseline characteristics and time-dependent risk factors

The default baseline characteristics used by the model are detailed in Table 1 and are consistent with profiles reported in the recent guideline for T1DM issued by the National Institute for Health and Care Excellence (NICE) [4]. The likelihood of clinical events is influenced by a number of risk factors that are time-dependent and, consistent with other models [13], we assume in the absence of any specific intervention that HbA1c will increase annually by 0.045% [29]. Furthermore, while the model has the capability of allowing other modifiable risk factors to change over time we hold weight, blood pressure and lipid parameters constant with respect to time for this study.

**Table 1:** Baseline cohort characteristics and model inputs.

**Variable**	**Mean**			**Standard Deviation**			**Source**
Age (years)	42.98			19.14			[4]
Duration (years)	16.92			13.31			
Proportion male	0.57			-			
HbA1c (%)	8.60			4.00			
SBP(mmHg)	128.27			16.07			
DBP(mmHg)	73.55			15.25			[30]
Total-C (mg/dL)	176.50			33			[4]
HDL-C (mg/dL)	50.25			13			
BMI (kg/m^2^)	27.09			5.77			
Proportion smoker	0.22			.			
NSHE	29			6.48			[31]
SHE	0.46			0.064			
**Event**	**Utility Decrement**	**Source**	**Event Cost (*£*)**	**SE**	**Maintenance Cost (*£*)**	**SE**	**Source**
Baseline	0.810	[32]	-	-	-	-	-
CVD (non-fatal)	-0.076^†^	[32, 33]	4688.69	468.87	585.75	58.57	[4]
CVD (fatal)	-		3824.34	382.43			
BDR	-*	[34]	-	-	-	-	
PDR	-0.086**		-	-	-	-	
Severe vision loss	-0.185***		5585	558.5	5396	540	
Macular edema	-	Assumed	-	-	-	-	
Micro-albuminuria	-	Assumed	-	-	-	-	
Macro-albuminuria	-0.017^‡^	[35]	-	-	-	-	
Impaired GFR	-0.017	Assumed					
Dialysis	-0.330	[32]	30480	3048	30480	3048	[4]
Transplant	-0.076	[36]	20373	2037.3	7609	760.9	
						
Neuropathy	-0.055	[35]	361.6	36.16	361.6	36.16	
Ketoacidosis	-	Assumed	952	95.2	-	-	
PVD	-	Assumed					
Uncomplicated FU	-0.083^✶^	[37]	4070	407	5483	54.83	[4]
Deep foot infection	-0.083	Assumed	7328	732.8	7328	732.8	
FU/critical ischaemia	-0.083	Assumed	10336	1.033.60	10336	1036.6	
Minor amputation	-0.116^ⅎ^	[35]	11290	1129	11290	1129	
Major amputation	-0.116		11290	1129	11290	1129	
NSHE^₸^	-0.014	[7]	-	-	-	-	
SHE₸	-0.047		333	-	-	-	[27]
BMI	-0.006	[38]	-	-	-	-	[4]
Hyperlipidaemia			38.22 -	3.82	38.22	3.82	
ACE inhibitor therapy			18.54	1.85	18.54	1.85	

ACE: angiotensin-converting-enzyme; BDR: background diabetic retinopathy; BMI: body mass index; C: cholesterol; CVD: cardiovascular disease; DBP: diastolic blood pressure; FU: foot ulcer; GFR: glomerular filtration rate; HbA1c: haemoglobin A1c; HDL: high-density lipoprotein; NSHE: nocturnal non-severe hypoglycaemic event; PDR: proliferative diabetic retinopathy; PVD: peripheral vascular disease; SBP: systolic blood pressure; SHE: severe hypoglycaemic event.

^†^ CVD was calculated as 60% MI, 32% angina and 8% stroke, where a utility decrement of 0.06 for MI and 0.22 for stroke were taken from Lung et al. A utility decrement of 0.07 for angina was taken from Lee et al.

* BDR taken as a 6/6–6/9 vision on the visual acuity scale.

** PDR taken as a 6/12–6/18 vision on the visual acuity scale.

*** Severe vision loss taken as 6/60–6/120 vision on the visual acuity scale.

^‡^ value was taken as diabetic kidney disease.

^✶^ value was taken as a generic ulcer, assumed equal for uncomplicated and complicated foot ulcer as well as foot ulcer with critical ischaemia.

^ⅎ^ value taken was for generic amputation, assumed equal for minor and major.

^₸^ Disutility presented as mean per event although the model implements the regression equations reported in [7] linking frequency and severity of hypoglycaemia to utility via the fear of hypoglycaemia score.

### Costs and utilities

The model considers the direct costs associated with the treatment and management of T1DM in addition to the costs associated with complications and adverse events. Complication related costs are partitioned into three components: fatal, non-fatal and maintenance costs. Fatal or non-fatal costs are applied within the cycle in which that event occurred. Maintenance costs for those surviving are applied in all subsequent years until either the subject dies or the simulated time horizon is reached. Table 1 reports UK specific costs (inflated to 2013/14 values using the PSSRU Hospital and Community Health Services (HCHS) index [39].

The occurrence of diabetes-related events is also associated with reduction in quality of life; the utility decrements used by the model are also reported in Table 1. The decrements applied are consistent with the default values used in the CORE Diabetes Model [40] and applied in recent guidelines [4]. Different disutility values may be specified for the year in which the event occurs and the years that follow. The model handles utility decrements for multiple events by applying the individual decrements additively. Disutility values should be entered in to the model as positive values.

### Parameter uncertainty

The output from individual patient level simulation models exhibit variability due to first-order uncertainty (random walk) and parameter (second-order) uncertainty. The model consequently simulates cohorts of up to 10,000 individuals to eliminate first-order uncertainty and the assessment of parameter uncertainty is undertaken by repeatedly simulating cohorts (up to 1,000) with input parameters for each cohort sampled from either normal (patient specific), gamma (costs) or beta (utilities) distributions. Due to the lack of published covariance information, sampled input data are independently generated. Uncertainty is quantified via cost-effectiveness acceptability curves and incremental cost-effectiveness ratio (ICER) scatterplots.

### Model validation

The availability of candidate external validation studies suitable for assessing the model’s predictive performance are relatively limited in T1DM; principally due to the key sources of epidemiological data (DCCT/EDIC) forming the basis of the model’s disease progression rates. The Supplementary Material (S1 Appendix) contains verification of the internal validation of the model’s equations to source data; we also present internal validation results for the model’s endpoint predictions when these equations are utilised within the model. To assess the external consistency of the model’s predictions we assessed the clinical events, costs and QALYs predicted by the model with a number of other new and established T1DM models; in particular: the Sheffield patient level simulation model [41]; the CRC discrete event simulation [42, 43]; the Treeage based model from McQueen et al. [44]; the patient level CORE Diabetes simulation model [45–48] and the patient-level simulation described by Wolowacz et al. [49]

In each case the CT1DM Model was initiated with baseline cohort, cost and health utility profiles consistent with those reported or cited in each publication and model output compared over the relevant time horizons. When comparing output, a number of candidate statistical tests for comparing model output with observed outcomes exist; however, there is little consensus upon the best approach [31]. Formal hypothesis testing is complicated by the fact that the disease model we are seeking to evaluate is only an approximation to the actual disease; consequently testing the null hypothesis of no difference between the validation study observation and model predictions makes little sense. However, to understand where model fit was poor, we also assessed goodness of fit between predicted (C1TDM Model) and observed (internal validation study endpoints and endpoints, costs and QALYs from other T1DM models) using the mean absolute percentage error (MAPE). These were calculated by comparing X (the predicted output) with Y (observed output): X1, X2, …, Xn and Y1, Y2, …, Yn where n is the sample size (the number of validation endpoints). We define the residuals Z as the paired difference between the two sets of results (predicted and observed): Z = Y − X, i = 1, 2, …, n. Calculation of the MAPE was computed using:

$$MAPE\;=\;\frac{1}{n}\overset{n}{\underset{i\;=\;1}{\sum }}\left|\left(\frac{Y_{i}-\;X_{i}}{Y_{i}}\right)\;x\;100\right|$$

Finally, and consistent with other validation studies published in the health economic literature, we present scatterplots of observed versus predicted endpoints along with the coefficient of determination (R^2^).

### Analysis

The analysis undertaken for this study utilised a simulated cohort of 10,000 individuals modelled over a 80 year time horizon. To ensure convergence of results each simulated cohort was replicated 1,000 times with all summary statistics and measures of precision relating to the mean and standard error of these 1,000 replicates. The model was initialised with a population profile consistent with recently published UK based clinical guidelines [4] as presented in Table 1; rates of hypoglycaemia were taken from the UK Hypoglycaemia Study Group [31]. Costs and health utility decrements associated with macro- and microvascular complications, hypoglycaemia and weight change were sourced from the published literature, as indicated in Table 1.

The model was used firstly to evaluate the benefit (measured by a change in predicted QALYs) associated with a 1% improvement in HbA1c. Subsequently, the following treatment related changes were applied to the baseline cohort profile: NSHE rates were modified by ±10%, ±20% or ±30%); weight was then modified by ±1kg, ±2kg or ±3kg). These changes were evaluated singularly and in combination. All changes were applied over the first 6 months and maintained for the patient’s lifetime; total and incremental QALYs evaluated over a 80-year horizon and discounted at 3.5% annually.

Secondly, the model was used to evaluate the impact of unit (%) changes in HbA1c on per-patient cost savings, QALY gains and therefore the health economic value (defined as the amount of additional spend (£) justified to obtain the additional QALY gain incorporating costs savings predicted for each unit reduction in HbA1c using a willingness-to-pay threshold of £20,000).

## Results

### Validation

**Fig 2:**
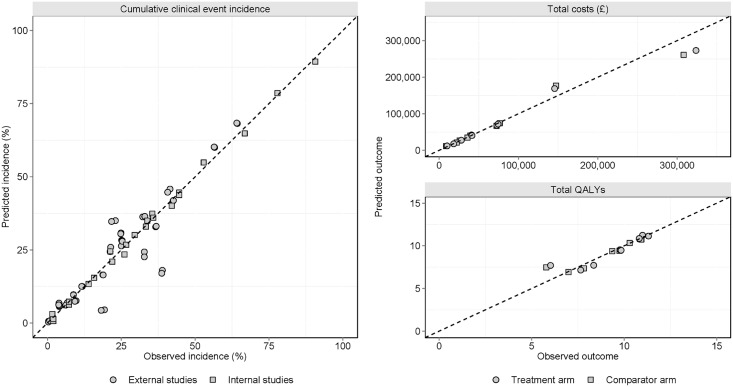
Model validation. Observed versus predicted validation endpoints (internal and external) and validation to published T1DM model output (costs and quality adjusted life years). Overall validation coefficient of determination for clinical endpoints, *R*^2^ = 0.863; internal *R*^2^ = 0.999; external *R*^2^ = 0.823; total costs *R*^2^ = 0.979; total QALYs *R*^2^ = 0.951.

Observed versus predicted validation endpoints, costs and QALYs are presented graphically in Fig 2. Overall, the validation coefficient of determination for clinical endpoints was R^2^ = 0.863 (internal R^2^ = 0.999; external R^2^ = 0.823) and total costs R^2^ = 0.979; total QALYs R^2^ = 0.951. Regression analysis indicated that endpoint predictions and costs had non-significant intercept terms (p = 0.009 and p = 0.652 respectively) indicating no systematic over or under-prediction. MAPE to predicted endpoints was 135.6% overall (11.3% internal and 213.6% external); MAPE for total costs was 26.0% and total QALYs was 21.0%.

### QALY gains associated with weight and hypoglycaemia

**Fig 3:**
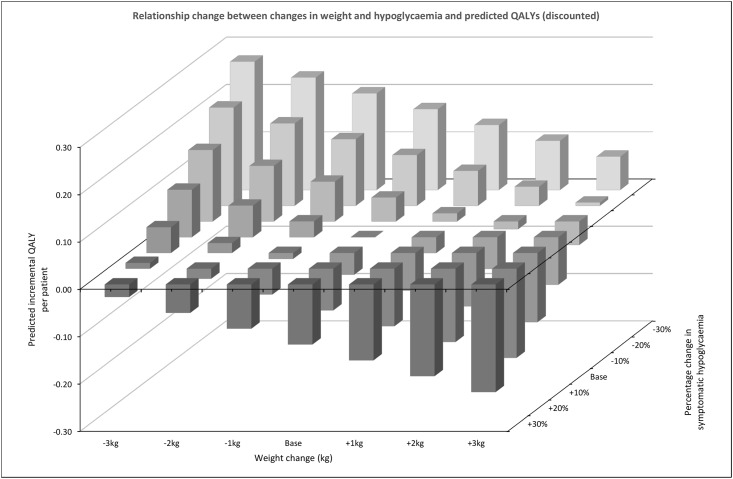
Weight and hypoglycaemia QALY plot. Assessing the impact of changes in weight and rates of hypoglycaemia events on per-patient lifetime quality-adjusted life year (QALY) difference. The reference point relates to a 1% reduction in HbA1c (%) with no associated changes in weight or hypoglycaemia, which was associated with a predicted QALY gains of 0.99. This figure illustrates the relative impact of weight change *±*3 kg and hypoglycaemia changes *±*30% on the QALY gained, beyond those already seen with the reference point.

Running the simulation model with the baseline cohort profiles specified in Table 1 resulted in a mean predicted life expectancy of 69.25 (conditional upon a start age of 42.98) representing 26.3 and 16.0 additional undiscounted life years and QALYs (16.8 and 10.4 discounted life years and QALYs respectively). Achieving and maintaining a 1% reduction in HbA1c was associated with an estimated gain of 0.64 and 0.61 discounted life years and QALYs respectively (the similarity in life years and QALY gains is driven principally by discounting). Changes in weight by ±1kg, ±2kg or ±3kg were associated with changes in discounted QALYs of ±0.03, ±0.07 and ±0.10 respectively. Modifying hypoglycaemia frequency by -10%, -20% or -30% resulted in changes to discounted QALYs of +0.05, +0.11 and +0.17 respectively. Modifying hypoglycaemia frequency by +10%, +20% or +30% resulted in changes to discounted QALYs of -0.05, -0.09 and -0.13 respectively. The combined effect of increasing weight by 3kg and a 30% increase in the frequency of hypoglycaemia reduced quality-adjusted life expectancy by 0.23, see Fig 3.

### The health economic value of improving glycaemic control

**Fig 4:**
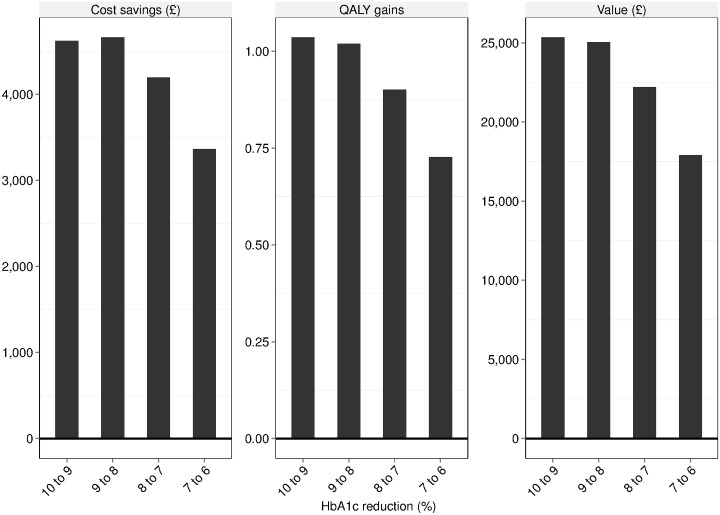
Health economic value associated with various levels of glucose control. Assessing the impact of unit (%) changes in HbA1c on per-patient cost savings, QALY gains and health economic value (defined as the amount of additional spend (*£*) justified to obtain the additional QALY gain predicted for each unit reduction in HbA1c at a willingness-to-pay threshold of *£*20, 000.

When initiated with cohort profiles specified in Table 1 the model estimates undiscounted lifetime per-patient costs of £72,586, 23.9 life-years and 14.0 QALYs (discounted values: £37,377; life expectancy of 15.8 years and QALYs of 9.5) at an HbA1c of 10%. When contrasted with the maintenance of HbA1c at 6% total undiscounted per-patient cost reduces to £39,508 (Δ = £33,078), 30.2 life years (Δ = 6.3) and 19.3 QALYs (Δ = 5.3). Discounted per-patient values were £18,340 (Δ = £19,037), 18.3 life years (Δ = 2.5) and 11.8 QALYs (Δ = 2.3). Fig 4 illustrates the impact of unit (%) changes in HbA1c on discounted per-patient cost savings, QALY gains and value; this plots highlights the greatest expected impact on costs, QALYs and consequently value is achieved with HbA1c reductions from 10% to 9% and 9%-8%.

## Discussion

The availability of long-term follow-up data from the original DCCT cohort in addition to the publication of T1DM specific cardiovascular risk equations has resulted in a renewed interest in the development and updating of T1DM health economic simulation models. The CT1DM Model was initially based on the original CORE Diabetes Model and the update described in this study is methodologically consistent with the approach taken by other modelling groups [17, 49, 50]. Consequently, the model is structurally consistent with other T1DM health economic models [15]. The model includes data drawn principally from the DCCT/EDIC and the Swedish National Diabetes Registry and the validation analysis presented indicates the model provides consistent predictions to both validation endpoints and cost-effectiveness output reported by other Type 1 diabetes models. The analysis presented in this study is also consistent with the research undertaken using the Cardiff Type 2 Diabetes Model in which key health economic issues related to diabetes have been assessed; for example, assessing the impact of risk factor changes on costs and outcomes [51]; the cost-effectiveness of treatment strategies at a population level [52]; the relative impact of weight, hypoglycaemia and HbA1c changes upon predicted QALYs [12] and the impact of variance reduction techniques on computation time [53].

Consistent with results reported in type 2 diabetes [12] this study highlights that the beneficial effects of improved glycaemic control on QALYs, achieved through the avoidance of diabetes-related complications, may be offset by characteristic treatment-specific adverse effects, such as weight gain and hypoglycaemia. The comparative weight and hypoglycaemic profiles of available therapies are therefore key to both their cost-effectiveness and effectiveness in clinical practice. Importantly, our evaluation is independent of any specific treatment and sought to quantify the value associated with attributes pertinent to the management of glucose control in T1DM, in particular, hypoglycaemia and weight change. We believe this to be an important consideration as it defines health economic value that is tailored to the patient profile rather than a specific therapeutic profile. As such, the health economic approach adopted here supports the ethos of personalized care and circumvents a key methodological challenge when evaluating competing technologies in T1DM; namely the synthesis of data across structurally heterogeneous clinical trials. For example, differing dose-titration algorithms, definitions of hypoglycaemia, variation in target levels and the number/timing of targets (for example, fasting blood glucose and/or post-prandial glucose) result in treatment effects that are specific to each individual trial. Our focus in this study seeks to provide an assessment of the health economic value of glucose lowering within the context of changes in hypoglycaemia frequency and weight change regardless of any specific particular intervention. We believe this approach offers an important complementary benefit over conventional analyses in that it seeks to quantify health economic value from the perspective of clinical outcomes achieved rather than an assessment of clinical and economic inputs.

Consistent with most modelling studies there are inevitably a number of important limitations with our model. We do not currently model recurrent CVD, which is reflective of a lack of relevant epidemiological data. However, it is unlikely that this omission will significantly influence the models results as the incidence of recurrent CVD in a type 2 diabetes population aged 40–97 years has been documented at a relatively low rate of 6% per year [54]. This has also been evaluated within the context of cost effectiveness in which the omission of subsequent events had no material impact upon predicted incremental cost effectiveness [55].

A further limitation relates to the analysis undertaken rather than the structural design of the model. While the model is capable of predicting time-dependent risk factor trajectories the analysis presented in this study has not sought to incorporate this feature. Our motivation for this was to ensure that we were able to present the marginal contributions to changes in costs and QALYs associated with changes in glycaemic control, weight and hypoglycaemia with all other factors held constant. Our analysis has sought to characterise the inter-relationship between changes in weight, hypoglycaemia and HbA1c and their individual impact upon life years and QALYs. In this application hypoglycaemia and weight are principally impacting health utility, while changes in HbA1c modifies the risk of complications and therefore influences both QALYS and life expectancy. A limitation of this analysis is that we do not quantify the inter-relationship between weight and blood pressure or cholesterol and therefore it is likely that the benefits of weight loss (or avoiding weight gain) are underestimated. Consequently, the interpretation of the analysis presented here should take this limitation into consideration, particularly as multifactorial risk factor management is a matter of routine clinical practice.

To conclude, we have presented an outcome-focused perspective to assessing the health economic consequences of differing levels of glycaemic control in T1DM with and without the effects of weight change and hypoglycaemia. The model reported uses contemporary data that enables the impact of a variety of risk factor management strategies on cost and outcomes to be assessed. Given the particular challenge that exists with respect to achieving optimal glucose control within the context of weight gain and hypoglycaemia acting as potential barriers this model provides an addition decision support tool for those seeking to ensure that current therapeutic approaches to the management of T1DM represent value for money.

## Supporting Information

S1 Appendix Supplementary Material. (DOCX)
