# Supplementary material for: The Health Economic Value of Changes in Glycaemic Control, Weight and Rates of Hypoglycaemia in Type 1 Diabetes Mellitus
Source: PLoS One. 2016 Sep 15;11(9):e0162441. doi: 10.1371/journal.pone.0162441 (PMC5025276; doi:10.1371/journal.pone.0162441)
Supplement: S1 Appendix — (DOCX) [file pone.0162441.s001.docx]

**S1 Appendix. Supplementary Material.**

**Table of Contents**

1 Health states 4

2 Predictive risk factors 4

3 Derivation of risk equations 5

3.1 Weibull hazard function 5

3.2 Natural progression of modifiable risk factors 6

3.3 Diabetic retinopathy and diabetic macular edema 7

3.4 Diabetic nephropathy 10

3.5 Diabetic neuropathy 16

3.6 Ketoacidosis 19

3.7 Cardiovascular disease 20

3.8 Hypoglycaemia 22

3.9 All-cause mortality 22

3.10 Summary of model equations and rates 23

4 References 25

**List of Tables**

Table 1: Summary of T1DM health states in the Cardiff Type 1 Diabetes Model 4

Table 2: Summary of patient characteristics that are predictive risk factors 4

Table 3: Diabetic retinopathy and macular edema event progression and their predictive risk factors.. 7

Table 4: Weibull parameters and risk factor coefficients for diabetic retinopathy and macular edema transitions. 8

Table 5: Diabetic nephropathy progression and their predictive risk factors. 12

Table 6: Weibull parameters and risk factor coefficients for diabetic nephropathy transitions. 12

Table 7: Static transition rates for diabetic nephropathy transitions. 16

Table 8: Diabetic ulcer and amputation progression and their predictive risk factors. 17

Table 9: Weibull parameters and risk factor coefficients for diabetic neuropathy transitions. 18

Table 10: Static transition rates for diabetic neuropathy transitions. 19

Table 11: Ketoacidosis progression and its predictive risk factors. 20

Table 12: Static transition rates for ketoacidosis. 20

Table 13: CVD progression and its predictive risk factors 21

Table 14: Coefficients for CVD risk equation (Eq. 12) 21

Table 15: Summary of equations and rates used within the model 23

**List of Figures**

Figure 1: Disease progression in those with diabetic retinopathy. 7

Figure 2: Fitting of incident BDR cumulative Weibull hazard to DCCT/EDIC data for intensive (INT) and conventional (CON) therapy. 9

Figure 3: Fitting of BDR to PDR cumulative Weibull hazard to DCCT/EDIC data for intensive (INT) and conventional (CON) therapy. 9

Figure 4: Fitting of incident ME cumulative Weibull hazard to DCCT/EDIC data for intensive (INT) and conventional (CON) therapy. 10

Figure 5: Disease progression in those with diabetic nephropathy. 11

Figure 6: Fitting of incident μAU cumulative Weibull hazard to DCCT/EDIC data for intensive (INT) and conventional (CON) therapy. 13

Figure 7: Fitting of μAU regression cumulative Weibull hazard to DCCT/EDIC data for intensive (INT) and conventional (CON) therapy. 14

Figure 8: Fitting of μAU to MAU cumulative Weibull hazard to DCCT/EDIC data for intensive (INT) and conventional (CON) therapy. 14

Figure 9: Fitting of μAU to iGFR cumulative Weibull hazard to DCCT/EDIC data for intensive (INT) and conventional (CON) therapy. 15

Figure 10: Fitting of MAU to ESRD cumulative Weibull hazard to DCCT/EDIC data for intensive (INT) and conventional (CON) therapy. 15

Figure 11: Disease progression in those with diabetic ulcer. 17

Figure 12: Fitting of incident DPN cumulative Weibull hazard to DCCT/EDIC data for intensive (INT) and conventional (CON) therapy 19

Figure 13: Disease progression in those with a ketoacidosis event 20

Figure 14: Disease progression in those with cardiovascular disease 20

Figure 15: Modelling of hypoglycaemia 22

# Health states

Health states in the Cardiff type 1 Diabetes Model are summarised in Table 1.

Table 1: Summary of T1DM health states in the Cardiff Type 1 Diabetes Model

| **Classification** | **Type 1 diabetes health states** | **Acronyms used in the Cardiff Type 1 Diabetes model** |
| --- | --- | --- |
| Retinopathy and macular edema | Background diabetic retinopathy | BDR |
|  | Proliferative diabetic retinopathy | PDR |
|  | Macular edema | ME |
|  | Severe vision loss | SVL |
| Nephropathy | Micro-albuminuria | μAU |
|  | Macro-albuminuria | MAU |
|  | Macro-albuminuria with impaired glomerular filtration rate | Impaired-GFR |
|  | Dialysis | - |
|  | Renal transplant | - |
| Neuropathy | Diabetic peripheral neuropathy | DPN |
|  | Uncomplicated foot ulcer | - |
|  | Deep foot infection | - |
|  | Foot ulcer and critical ischaemia | - |
|  | Minor amputation | - |
|  | Major amputation | - |
| Ketoacidosis | Ketoacidosis | - |
| Cardiovascular disease | Cardiovascular disease | CVD |
|  | Subsequent CVD | Subsequent CVD |

# Predictive risk factors

Patient characteristics that influence the likelihood of clinical events (predictive risk factors) are detailed in Table 2.

Table 2: Summary of patient characteristics that are predictive risk factors

| **Patient characteristic** | **Description** | **BL *** | **TE †** | **RF ⱡ** | **Units** |
| --- | --- | --- | --- | --- | --- |
| **Demographics** | | | | | |
| Age | The age of the subject | Y | N | Y | Years |
| Age at diabetes onset | Age at diagnosis of T1DM | N | N | N | Years |
| Gender | The proportion of the cohort that are male/female  (1 = female, 0 = male) | Y | N | N | Proportion |
| Height | Subject height | Y | N | N | m |
| Smoke | Subject smoking status (1 = smoker, 0 = non-smoker) | Y | N | N | Proportion |
| **Modifiable risk factors** | | | | | |
| HbA1c | HbA1c measurement | Y | Y | Y | % |
| Weight | Subject weight | N | Y | Y | Kg |
| BMI | Body mass index | N | Y | Y | Kg/m^2^ |
| TC | Total cholesterol | Y | Y | N | mg/dL |
| HDL | High density lipoprotein cholesterol | Y | Y | N | mg/dL |
| SBP | Subject systolic blood pressure | Y | Y | N | mmHg |
| DBP | Subject diastolic blood pressure | Y | Y | N | mmHg |
| MAP | Subject mean arterial pressure | Y | Y | N | mmHg |
| **Event/treatment history**** | | | | | |
| Hyperlipidaemia | TC/HDL ratio > 4.5 or on lipid-lowering agent | Y | N | N | Proportion |
| RAASi therapy | On renin-angiotensin-aldosterone system inhibitors | Y | N | N | Proportion |
| Hypertension | SBP > 140 mmHg or DBP > 90 mmHg | Y | N | N | Proportion |
| Micro-albuminuria | Has micro-albuminuria | Y | N | Y | Proportion |
| Macro-albuminuria | Has macro-albuminuria | Y | N | Y | Proportion |
| CVD | In CVD state | Y | N | Y | Proportion |
| Previous ulcer | Has had a previous ulcer | Y | N | Y | Proportion |
| Minor amputation | Has had minor amputation | Y | N | Y | Proportion |
| Major amputation | Has had major amputation | Y | N | Y | Proportion |
| PVD | History of PVD |  |  |  | Proportion |
| **Event/treatment history characteristics** | | | | | |
| Duration diabetes | Time since diabetes diagnosis | Y | N | Y | Years |
| Duration BDR | If subject has BDR, years since onset | Y | N | Y | Years |
| Duration micro AU | If subject has micro-albuminuria, years since onset | Y | N | Y | Years |
| Duration macro AU | If subject has macro-albuminuria, years since onset | Y | N | Y | Years |
| Duration treatment | Years on current treatment line | Y | N | Y | Years |
| Duration dialysis | If subject in ESRD and receiving dialysis, years since dialysis initiation | Y | N | Y | Years |

BMI: body mass index; BDR: background diabetic retinopathy; CVD: cardiovascular disease; DBP: diastolic blood pressure; DPN: diabetic peripheral neuropathy; ESRD: end-stage renal disease; HbA1C: haemoglobin A1C; HDL-C: high-density lipoprotein cholesterol; LDL-C: low-density lipoprotein cholesterol; MAP: mean arterial pressure; PVD: peripheral vascular disease; RAASi: renin-angiotensin-aldosterone system inhibitor; SBP: systolic blood pressure; T1DM: type 1 diabetes mellitus; TC: total cholesterol.

* BL: Configurable at baseline [Y/N]

† TE: Modified by treatment effect [Y/N]

ⱡ RF: Modified due to risk factor progression or event incidence [Y/N]

**For event/treatment history 1 = in health sate or with treatment history, 0 = not in event state or no treatment history.

# Derivation of risk equations

The risk equations for the prediction of retinopathy and macular edema, nephropathy and peripheral neuropathy were developed to fit data from the DCCT and EDIC studies.^1-4^ The DCCT involved 1,441 volunteers, aged 13 to 39, with T1DM at 29 medical centres in the United States and Canada. When the DCCT ended in 1993, researchers continued to study more than 90% of participants in the EDIC study, and assessed the incidence and predictors of CVD events, as well as diabetic complications related to the eye (retinopathy), kidney (nephropathy), and nerves (neuropathy). Where DCCT/EDIC data did not allow for the development of risk equations, alternative data sources/risk equations were used instead.^5-9^

## Weibull hazard function

The risk associated with time-dependent transitions is modelled using a Weibull hazard, the form of which is:

$$h\left( t | Z\left( t \right) \right)=\left( \frac{k}{\lambda_{0}} \right)\left( \frac{t}{\lambda_{0}} \right)^{k-1}e^{{Z(t)}^{T}\beta}.$$

The cumulative hazard, which is comparable with the cumulative incidence, is given by the integral:

$$H\left( t | Z\left( t \right) \right)= \int_{0}^{t} h\left( s | Z\left( s \right) \right) ds\approx\left( \frac{t}{\lambda_{0}} \right)^{k}e^{{Z\left( t \right)}^{T}\beta} ,$$

where the approximation is exact when the covariates, $Z\left( t \right)$, do not change in time. Given these, the probability of an event occurring within a given time interval $(t,t+\tau]$ is:

$$P\left( t<T_{\text{event}}\leq t+\tau| t<T_{\text{event}}, Z\left( t \right) \right)= exp\left( H\left( t+\tau| Z\left( t+\tau\right) \right)-H\left( t+\tau| Z\left( t+\tau\right) \right) \right)\approx exp\left( \left( \left( \frac{t+\tau}{\lambda_{0}} \right)^{k}-\left( \frac{t}{\lambda_{0}} \right)^{k} \right)e^{{Z\left( t \right)}^{T}\beta} \right),$$

which is used in the model to determine whether a given transition happens.

In order to derive the parameters for such hazard curves, cumulative incidence curves from each respective study were read digitally using engauge^10^ (a digital transcription tool). Data relating to potential risk factors such as HbA1c, blood pressure, cholesterol and duration of diabetes were similarly transcribed from additional studies describing the follow up of patients over the studies duration. ^2, 11^ Utilising the cumulative incidence data, Weibull curves were fitted, assessing for any interaction between relevant risk factors.

## Natural progression of modifiable risk factors

The likelihood of clinical events occurring is influenced by risk factors. Some risk factors, namely HbA1c, DBP, SBP, TC, HDL-C, weight and MAP, are modifiable by treatment effects (as described in section 6). Additionally, HbA1c and weight may follow user-specifiable time-dependent trajectories. As the trajectory of such risk factors change so does their influence upon the likelihood of an event occurring.

The natural history progression of HbA1c and weight is modelled via annual user-defined rates. For example, an annual weight gain of 0.1kg may be applied, resulting in a weight gain of 1kg over a 10-year period (assuming there are no additional therapy effects).

Under default settings the annual weight gain is 0.1kg, whilst HbA1c is assumed to increase annually at a rate of 0.045%.^12^ The natural history progression of DBP, SBP, TC HDL-C and MAP, unless a treatment effect is incurred, are assumed to remain constant over the modelled time horizon. The risk equations for the prediction of retinopathy and macular edema, nephropathy and peripheral neuropathy were developed to fit data from the DCCT and EDIC studies.^1-4^ The DCCT involved 1,441 volunteers, aged 13 to 39, with T1DM at 29 medical centres in the United States and Canada. When the DCCT ended in 1993, researchers continued to study more than 90% of participants in the EDIC study, and assessed the incidence and predictors of CVD events, as well as diabetic complications related to the eye (retinopathy), kidney (nephropathy), and nerves (neuropathy). Where DCCT/EDIC data did not allow for the development of risk equations, alternative data sources/risk equations were used instead.^5-9^

## Diabetic retinopathy and diabetic macular edema

Diabetic retinopathy and diabetic macular edema (ME) are key microvascular complications in T1DM patients and may have a sudden and debilitating impact on visual acuity eventually leading to severe vision loss (SVL) or blindness.


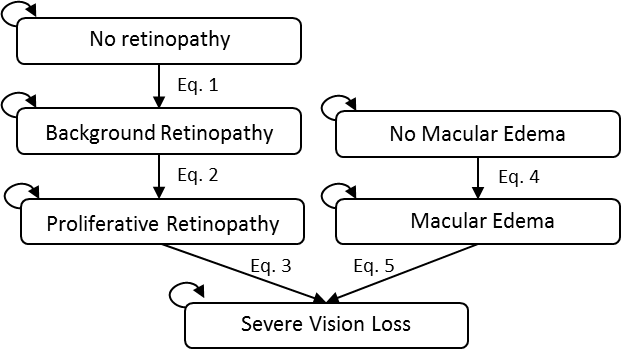


Figure 1: Disease progression in those with diabetic retinopathy.

Patients may progress from no retinopathy to background diabetic retinopathy (BDR), to proliferative diabetic retinopathy (PDR) and to severe vision loss (SVL), (see Figure 1). Patients with diabetic ME may progress from no ME to ME and to SVL, Figure 1. Assigned equations used in diabetic retinopathy and ME disease progression, and their predictive risk factors are summarised in Table 3.

Table 3: Diabetic retinopathy and macular edema event progression and their predictive risk factors..

| **Progression** | **Risk factors** | **Equation number** | **Source** |
| --- | --- | --- | --- |
| Incident BDR | Duration of diabetes, HbA1c, proportion with hyperlipidemia, proportion with micro-albuminuria, proportion current smoker, MAP, RAASi therapy | Eq. 1 | DCCT/EDIC^2^ |
| BDR to PDR | Duration of diabetes, HbA1c, proportion with hyperlipidemia, proportion with micro-albuminuria, MAP | Eq. 2 |  |
| PDR to SVL | HbA1c | Eq. 3 |  |
| Incident ME | Duration of diabetes, HbA1c | Eq. 4 |  |
| ME to SVL | HbA1c | Eq. 5 |  |

BDR: background diabetic retinopathy; CON: conventional therapy; Eq: equation; HbA1c: haemoglobin A1c; MAP: mean arterial pressure; ME: macular edema; PDR: proliferative diabetic retinopathy; RAASi: renin-angiotensin-aldosterone system inhibitor; SVL severe vision loss; μAU: micro-albuminuria.

Risk equations for diabetic retinopathy and ME event prediction were derived from DCCT/EDIC data.^2^ Fitting parameters for PDR to SVL and ME to SVL were taken from values given by Javitt et al.^13^ The Weibull parameters and risk factor coefficients are tabulated in Table 4. The derived cumulative hazard curves for incident BDR, BDR to PDR and incident ME are plotted against the data in Figure 2, Figure 3, Figure 4 respectively.

Table 4: Weibull parameters and risk factor coefficients for diabetic retinopathy and macular edema transitions.

| **Equation** | **Eq. 1** | **Eq. 2** | **Eq. 3** | **Eq. 4** | **Eq. 5** |
| --- | --- | --- | --- | --- | --- |
| Model Section | Diabetic Retinopathy | | | Diabetic Macular Edema | |
| Disease progression | Incident BDR | BDR to PDR | PDR to SVL | Incident ME | ME to SVL |
| Functional form | Weibull | Weibull | Weibull | Weibull | Weibull |
| Coefficient [reference value] | | | | | |
| *t* variable | Diabetes Duration | Years since BDR | N/A | Diabetes Duration | N/A |
| λ | 9.711 | 158.882 | 111.11 | 76.843 | 58.82 |
| *k* | 0.42 | 1.21 | 1.00 | 1.842 | 1.00 |
| HbA1c (centred on 7.07) | 0.392 | 0.615 |  | 0.627 |  |
| Smoker | 0.293 |  |  |  |  |
| RAASi^1^ | -0.041 |  |  |  |  |
| Hyperlipidaemia | 0.329 | 0.329 |  |  |  |
| μAU | 0.582 | 0.932 |  |  |  |
| MAP (centred on 85) | 0.020 | 0.039 |  |  |  |
| SBP (centred on 120) |  |  |  |  |  |
| DBP (centred on 80) |  |  |  |  |  |
| Laser therapy^ |  |  | -1.631 |  | -0.405 |
| Exposure to CON* |  | 0.648 |  | 0.456 |  |

BDR: background diabetic retinopathy; CON: conventional therapy; DBP: diastolic blood pressure; DPN: diabetic peripheral neuropathy; Hba1c: haemoglobin A 1c; MAP: mean arterial pressure; PDR: proliferative diabetic retinopathy; SBP: systolic blood pressure; SVL: severe vision loss.

Figure 2: Fitting of incident BDR cumulative Weibull hazard to DCCT/EDIC data for intensive (INT) and conventional (CON) therapy.

Figure 3: Fitting of BDR to PDR cumulative Weibull hazard to DCCT/EDIC data for intensive (INT) and conventional (CON) therapy.

Figure 4: Fitting of incident ME cumulative Weibull hazard to DCCT/EDIC data for intensive (INT) and conventional (CON) therapy.

## Diabetic nephropathy

Diabetic nephropathy is a microvascular complication in T1DM patients and is a leading cause of end-stage renal disease (ESRD) for which treatment is dialysis or renal transplant.

Patients progress from no nephropathy to micro-albuminuria, from which they may either return to no nephropathy or progress to macro-albuminuria with or without impaired glomerular filtration rate (GFR). Once in a state of macro-albuminuria (with or without impaired-GFR), patients may progress to a state of ESRD which is comprised of dialysis and renal transplant; currently patients who progress to ESRD state are split between the two states upon incidence; 89.9% receive dialysis and 10.1% receive a transplant.^8^


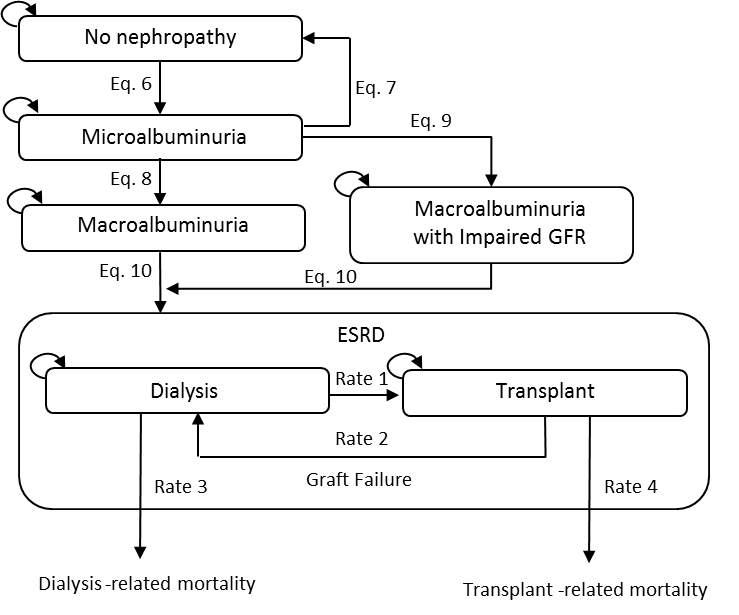


Figure 5: Disease progression in those with diabetic nephropathy.

Note: While the model contains functionality for patients with impaired GFR to be at an increased risk of progression to ESRD the model currently employs the same equation as patients progressing from macro-albuminuria. This will be updated upon the release of appropriate data.

Patients receiving dialysis treatment may undergo a kidney transplant, assuming they have not had a previous transplant. The rate at which patients progress from dialysis to transplant is informed by data from the UK Renal Registry which estimates an annual transplant incidence of 4.57%.^8^ It is assumed that patients may only undergo one transplant operation.

Similarly, a patient may return to dialysis treatment from the transplant state due to transplant graft failure. The rate (3.97% annually) at which this occurs is based on a contemporary study by Taber *et al*.^9^ that presents 5 year overall and graft survival in diabetic patients with a kidney transplant. Those in the ESRD health state (dialysis and transplant) are at increased risk of mortality.

Costs and utilities are applied to those in the micro-albuminuria, macro-albuminuria with impaired-GFR, macro-albuminuria, dialysis or transplant health states.

The assigned equation number used in diabetic nephropathy disease progression and their predictive risk factors are summarised in Table 5.

Table 5: Diabetic nephropathy progression and their predictive risk factors.

| **Progression** | **Risk factors** | **Equation number** | **Source** |
| --- | --- | --- | --- |
| Incident micro-albuminuria | Duration of diabetes, HbA1c, micro-albuminuria | Eq. 6 | De Boer *et al.*^3^ |
| Micro-albuminuria to normo-albuminuria | Duration of diabetes, duration of micro-albuminuria, HbA1c, DBP | Eq. 7 | De Boer *et al.^4^* |
| Micro-albuminuria to macro-albuminuria | Duration of diabetes, duration of micro-albuminuria, HbA1c, SBP, DBP | Eq. 8 | De Boer *et al.^4^* |
| Micro-albuminuria to macro-albuminuria with impaired-GFR | Duration of diabetes, duration of micro-albuminuria, HbA1c, SBP, DBP | Eq. 9 | De Boer *et al.^4^* |
| Macro-albuminuria to ESRD | Duration of macro-albuminuria | Eq. 10 | De Boer *et al.^4^* |
| Macro-albuminuria with impaired-GFR to ESRD | Duration of macro-albuminuria | Eq. 10. | De Boer *et al.^4^* |
| Dialysis to transplant | Not applicable | Rate 1 | UK Renal Registry^8^ |
| Transplant to dialysis (graft failure) | Not applicable | Rate 2 | Taber *et al.^9^* |
| Dialysis to death | Not applicable | Rate 3 | UK Renal Registry^14^ |
| Transplant to death | Not applicable | Rate 4 | Taber *et al.^9^* |

ESRD: end-stage renal disease; DBP: diastolic blood pressure; GFR: glomerular filtration rate; HbA1c: haemoglobin A1c; MAU: macro-albuminuria; μAU: Micro-albuminuria; SBP: systolic blood pressure.

Risk equations for diabetic nephropathy event prediction were either derived from DCCT/EDIC data^3, 4^, or using static transition rates derived from either a study by Taber *et al*.^9^, or derived from UK Renal Registry data.^8, 14^ The Weibull parameters and risk factor coefficients are tabulated in Table 4. The derived cumulative hazard curves for incident μAU, μAU regression, μAU to MAU, μAU to iGFR and MAU to ESRD are plotted against the data in Figure 6, Figure 7, Figure 8, Figure 9 and

Figure 10 respectively. Static transition rates for nephropathy transitions are tabulated in

Table 6: Weibull parameters and risk factor coefficients for diabetic nephropathy transitions.

| **Equation** | **Eq. 6** | **Eq. 7** | **Eq. 8** | **Eq. 9** | **Eq. 10** |
| --- | --- | --- | --- | --- | --- |
| Model Section | Diabetic Nephropathy | | | | |
| Disease progression | Incident μAU | μAU to Normo | μAU to MAU | μAU to MAU with Impaired GFR | MAU to ESRD |
| Functional form | Weibull | Weibull | Weibull | Weibull | Weibull |
| Coefficient [reference value] |  | | | | |
| *t* variable | Diabetes Duration | Years since μAU | Years since μAU | Years since μAU | Years since μAU |
| λ | 104.261 | 20.031 | 31.672 | 50.319 | 43.332 |
| *k* | 0.951 | 0.820 | 0.804 | 0.860 | 2.178 |
| HbA1c (centred on 7.07) | 0.514 | -0.236 | 0.223 | 0.120 |  |
| Smoker |  |  |  |  |  |
| RAASi^1^ |  |  |  |  |  |
| Hyperlipidaemia |  |  |  |  |  |
| μAU |  |  |  |  |  |
| MAP (centred on 85) |  |  |  |  |  |
| SBP (centred on 120) |  |  | 0.054 | 0.065 |  |
| DBP (centred on 80) |  | -0.039 | 0.071 | 0.077 |  |
| Laser therapy^ |  |  |  |  |  |
| Exposure to CON* |  | -0.358 | 0.557 | 0.557 | 0.165 |

CON: conventional therapy; DBP: diastolic blood pressure; DPN: diabetic peripheral neuropathy; ESRD: end-stage renal disease; GFR: glomerular filtration rate; Hba1c: haemoglobin A 1c; MAP: mean arterial pressure; μAU: Micro-albuminuria; MAU: Macro-albuminuria; SBP: systolic blood pressure.

Figure 6: Fitting of incident μAU cumulative Weibull hazard to DCCT/EDIC data for intensive (INT) and conventional (CON) therapy.

Figure 7: Fitting of μAU regression cumulative Weibull hazard to DCCT/EDIC data for intensive (INT) and conventional (CON) therapy.

Figure 8: Fitting of μAU to MAU cumulative Weibull hazard to DCCT/EDIC data for intensive (INT) and conventional (CON) therapy.

Figure 9: Fitting of μAU to iGFR cumulative Weibull hazard to DCCT/EDIC data for intensive (INT) and conventional (CON) therapy.

Figure 10: Fitting of MAU to ESRD cumulative Weibull hazard to DCCT/EDIC data for intensive (INT) and conventional (CON) therapy.

Table 7: Static transition rates for diabetic nephropathy transitions.

| **Equation** | **Rate 1** | **Rate 2** | **Rate 3** | **Rate 4** |
| --- | --- | --- | --- | --- |
| Model Section | Diabetic Nephropathy | | | |
| Disease progression | Dialysis to transplant | Transplant to dialysis (graft failure) | Dialysis to death | Transplant to death |
| Functional form | Static transition rate | Static transition rate | Static transition rate | Static transition rate |
| Rate | 4.57% | 3.97% | 16.723% | 3.73% |

## Diabetic neuropathy

Peripheral neuropathy in T1DM patients occurs as a result of high blood glucose levels damaging the peripheral nerves and most commonly presents as pain or numbness in the extremities (most often legs and feet). Damage to peripheral nerves usually occurs as a consequence of peripheral vascular disease (PVD), where narrowing of blood vessels in the peripheral vascular system can restrict blood flow, reducing the oxygen supply to the nerves resulting in damage. Damage to the peripheral nerves and micro-vasculature contributes to the development of diabetic ulcer, particularly of the foot, which a major compilation that can lead to minor or major amputation.

Patients may progress from no DPN to DPN with no ulcer. Only patients with DPN with no ulcer may progress to ulcer and amputation states. Patients with DPN with no ulcer can progress to uncomplicated foot ulcer, foot ulcer and critical ischaemia or deep foot infection. Patients in these three health states can also return to a state of no foot complications/no ulcer. If patients are in the foot ulcer and critical ischaemic or deep foot infection health state they may progress to either minor amputation, defined as amputation below the ankle, or major amputation, defined as amputation above the ankle.^5^


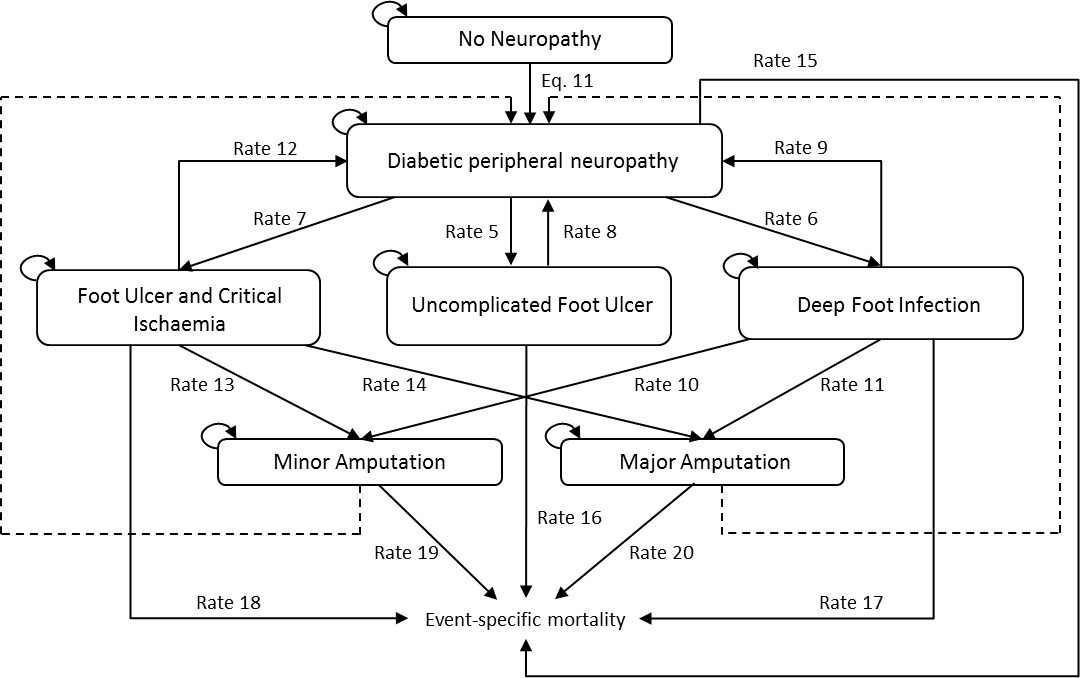


Figure 11: Disease progression in those with diabetic ulcer.

Patients in the uncomplicated foot ulcer, foot ulcer and critical ischaemia, deep foot infection, minor amputation or major amputation are at increased risk of mortality.^5^

Costs and utilities are applied to DPN with no ulcer, uncomplicated foot ulcer, foot ulcer and critical ischaemia, deep foot infection, minor amputation and major amputation health states.

The risk equation for incident diabetic peripheral neuropathy event prediction was derived from DCCT/EDIC data.^1^ Ulcer and amputation event predictions were derived from a study by Tennvall and Apelqvist.^5^

**Note:** Patients with minor and major amputation events return to a state of DPN with no ulcer but still incur maintenance and utility decrements associated with their amputation events. This is indicated by the dashed line in Figure 11.

The assigned equation number used in diabetic ulcer and amputation disease progression and their predictive risk factors are summarised in Table 8.

The annual transition rates used to inform the progression through neuropathy states are taken from a comprehensive Markov-state transition model described in the study by Tennvall and Apleqvist.^5^ Transition rates between each state are presented in Table 10 and are stratified in to four risk categories:

Risk group 1: No specific risk factors

Risk group 2: Patients with DPN (assumed to be a proxy for sensory neuropathy, as detailed in the original study)

Risk group 3: Patients with DPN (assumed to be a proxy for sensory neuropathy, as detailed in the original study) and PVD

Risk group 4: Patients with a history of ulcer or amputation

Table 8: Diabetic ulcer and amputation progression and their predictive risk factors.

| Progression | Risk factors | Eq. number | Source |
| --- | --- | --- | --- |
| No neuropathy to diabetic peripheral neuropathy | Duration of diabetes, HbA1c, SBP | Eq. 11 | Martin *et al.^1^* |
| Diabetic peripheral neuropathy to uncomplicated foot ulcer | PVD, previous ulcer or amputation* | Rate 5 | Tennvall and Apelqvist^5^ |
| Diabetic peripheral neuropathy to deep foot infection |  | Rate 6 |  |
| Diabetic peripheral neuropathy to foot ulcer and critical ischaemia |  | Rate 7 |  |
| Uncomplicated foot ulcer to diabetic peripheral neuropathy |  | Rate 8 |  |
| Deep foot infection to diabetic peripheral neuropathy | Not applicable | Rate 9 |  |
| Deep foot infection to minor amputation |  | Rate 10 |  |
| Deep foot infection to major amputation |  | Rate 11 |  |
| Foot ulcer and critical ischaemia to diabetic peripheral neuropathy |  | Rate 12 |  |
| Foot ulcer and critical ischaemia to minor amputation |  | Rate 13 |  |
| Foot ulcer and critical ischaemia to major amputation |  | Rate 14 |  |
| Diabetic peripheral neuropathy to death | PVD, previous ulcer or amputation* | Rate 15 |  |
| Uncomplicated foot ulcer to death |  | Rate 16 |  |
| Deep foot infection to death | Not applicable | Rate 17 |  |
| Foot ulcer and critical ischaemia to death |  | Rate 18 |  |
| Minor amputation to death |  | Rate 19 |  |
| Major amputation to death |  | Rate 20 |  |

AMP: amputation; DPN: diabetic peripheral neuropathy; PVD: peripheral vascular disease

*Previous ulcer or amputation include: previous ulcer, previous minor amputation, or previous major amputation.

Table 9: Weibull parameters and risk factor coefficients for diabetic neuropathy transitions.

| **Equation** | **Eq. 11** |
| --- | --- |
| Model Section | Diabetic Neuropathy |
| Disease progression | Incident DPN |
| Functional form | Weibull |
| *t* variable | Diabetes Duration |
| λ | 69.244 |
| *k* | 1.344 |
| HbA1c (centred on 7.07) | 0.560 |
| Smoker |  |
| RAASi^1^ |  |
| Hyperlipidaemia |  |
| μAU |  |
| MAP (centred on 85) |  |
| SBP (centred on 120) | 0.012 |
| DBP (centred on 80) |  |
| Laser therapy^ |  |
| Exposure to CON* | 0.165 |

CON: conventional therapy; DBP: diastolic blood pressure; DPN: diabetic peripheral neuropathy; Hba1c: haemoglobin A 1c; MAP: mean arterial pressure; SBP: systolic blood pressure.

Figure 12: Fitting of incident DPN cumulative Weibull hazard to DCCT/EDIC data for intensive (INT) and conventional (CON) therapy

Table 10: Static transition rates for diabetic neuropathy transitions.

| **Initial state** | **Subsequent state** | **Equation no.** | **Risk group 1** | **Risk group 2** | **Risk group 3** | **Risk group 4** |
| --- | --- | --- | --- | --- | --- | --- |
| No foot ulcer | Uncomplicated foot ulcer | Rate 5 | 0.0015 | 0.0220 | 0.0170 | 0.0970 |
|  | Deep foot infection | Rate 6 | 0.0000 | 0.0140 | 0.0110 | 0.0440 |
|  | Foot ulcer and critical ischaemia | Rate 7 | 0.0000 | 0.0000 | 0.0080 | 0.0290 |
|  | Death | Rate 15/ Rate 19/ Rate 20 | 0.0070 | 0.0175 | 0.0175 | 0.0270/0.1200 |
| Uncomplicated foot ulcer | No foot ulcer | Rate 8 | 0.9930 | 0.9825 | 0.9825 | 0.973 |
|  | Death | Rate 16 | 0.0070 | 0.0175 | 0.0175 | 0.0270 |
| Deep foot infection | No foot ulcer | Rate 9 | 0.4000 | 0.4000 | 0.4000 | 0.4000 |
|  | Minor amputation | Rate 10 | 0.3500 | 0.3500 | 0.3500 | 0.3500 |
|  | Major amputation | Rate 11 | 0.0900 | 0.0900 | 0.0900 | 0.0900 |
|  | Death | Rate 17 | 0.1600 | 0.1600 | 0.1600 | 0.1600 |
| Foot ulcer and critical ischaemia | No foot ulcer | Rate 12 | 0.3800 | 0.3800 | 0.3800 | 0.3800 |
|  | Minor amputation | Rate 13 | 0.1300 | 0.1300 | 0.1300 | 0.1300 |
|  | Major amputation | Rate 14 | 0.2700 | 0.2700 | 0.2700 | 0.2700 |
|  | Death | Rate 18 | 0.2200 | 0.2200 | 0.2200 | 0.2200 |

## Ketoacidosis

Diabetic ketoacidosis is a serious complication of diabetes that can occur when the body produces high levels of ketones. Insulin plays a key role in the use of sugar (glucose) as an energy source. Without enough insulin the body begins to use fat as an alternative energy source. This process can result in the build-up of acids, called ketones, in the bloodstream and can lead to diabetic ketoacidosis if left untreated.


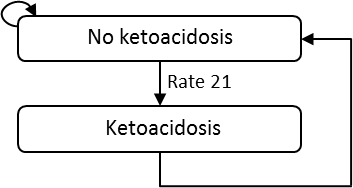


Figure 13: Disease progression in those with a ketoacidosis event

Table 11: Ketoacidosis progression and its predictive risk factors.

| **Progression** | **Risk factors** | **Equation number** | **Source** |
| --- | --- | --- | --- |
| Incident Ketoacidosis | Not applicable | Rate 21 | Wang *et al*.^7^ |

Ketoacidosis is modelled as an acute event health state. Patients may have a ketoacidosis event during any cycle of the modelled time horizon and may have multiple events over a lifetime. The rate of ketoacidosis incidence 1.585 per 100,000 persons is taken from a study by Wang *et al.^7^.*

Table 12: Static transition rates for ketoacidosis.

| **Equation** | **Rate 21** |
| --- | --- |
| Model Section | Ketoacidosis |
| Disease progression | Incident Ketoacidosis |
| Functional form | Static transition rate |
| Rate | 1.585 events per 100,000 patient years |

## Cardiovascular disease

Cardiovascular disease (CVD) is a term used to describe diseases that involve the heart or the brain. In this instance, cardiovascular disease is defined as incident myocardial infarction (MI), stroke or CVD-related death.


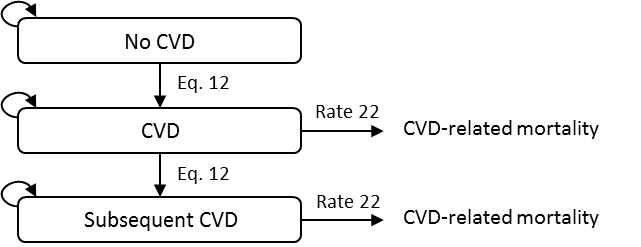


Figure 14: Disease progression in those with cardiovascular disease

Patients with no CVD can progress to a state of CVD. Once in the state of CVD, the may incur subsequent CVD events. The risk of CVD and subsequent CVD is modelled using a risk derived form a study by Cederholm *et al.^6^* .

Table 13: CVD progression and its predictive risk factors

| **Progression** | **Risk factors** | **Equation number** | **Source** |
| --- | --- | --- | --- |
| Incident CVD | Age at diabetes onset, duration of diabetes, TC/HDL ratio, HbA1c, SBP, BMI, gender, proportion current smoker, micro-albuminuria, macro-albuminuria, CVD history | Eq.12 | Cederholm *et al.^6^* |
| CVD to Subsequent CVD |  |  |  |
| CVD to death | Not applicable | Rate 22 | Rawshani *et al.^15^* |
| Subsequent CVD to death |  |  |  |

CVD: cardiovascular disease.

The risk of initial and subsequent CVD events is derived from a study by Cederholm et al.^6^, which reports a risk equation from the prediction of initial and subsequent CVD events derived from Swedish National Diabetes Registry (NDR) data.^16^ The coefficients of the risk equation are presented in Table 14. The form of the risk equation utilised to estimate the 5-year risk of CVD is given by:

$$5 year risk of CVD =1 -exp(-\left( \lambda\times\beta_{1}^{Age at diabetes onset}\times\beta_{2}^{Sex}\times\beta_{3}^{Duration of diabetes}\times\beta_{4}^{HbA1c}\times\beta_{5}^{BMI}\times\beta_{6}^{Hypertension}\times\beta_{7}^{SBP}\times\beta_{8}^{Lipid-lowering drugs}\times\beta_{9}^{Smoking status} \right))\times100$$

Table 14: Coefficients for CVD risk equation (Eq. 12)

| **Coefficient** | **Value** |
| --- | --- |
| λ | 0.00013 |
| *Age at diabetes onset (β_1_)* | 1.066 |
| Sex *(β_2_)* | 1.538 |
| Duration of diabetes *(β_3_)* | 1.087 |
| HbA1c *(β_4_)* | 1.117 |
| BMI *(β_5_)* | 1.017 |
| Hypertension *(β_6_)* | 1.278 |
| SBP *(β_7_)* | 1.007 |
| Lipid-lowering drugs *(β_8_)* | 1.314 |
| Smoking status *(β_9_)* | 1.492 |

Table 15: Static transition rates for CVD.

| **Equation** | **Rate 22** |
| --- | --- |
| Model Section | Cardiovascular disease |
| Disease progression | CVD-related mortality |
| Functional form | Static transition rate |
| Rate | Rate of 39.19% applied to all incident events |

## Hypoglycaemia

Hypoglycaemia is a potentially dangerous adverse event of therapy in diabetes and is modelled as an adverse event of therapy according to therapy-specific incidence rates.

Hypoglycaemia is separated into non-severe and severe events. The proportion of subjects that experience a severe event is defined by a user specified probability. Non-severe events are separated into user defined annual numbers of symptomatic and nocturnal hypoglycaemic events, Figure 15. Hypoglycaemia events incur a one off cost and utility decrement during the cycle in which they occurred. These events are user-defined and therapy specific.


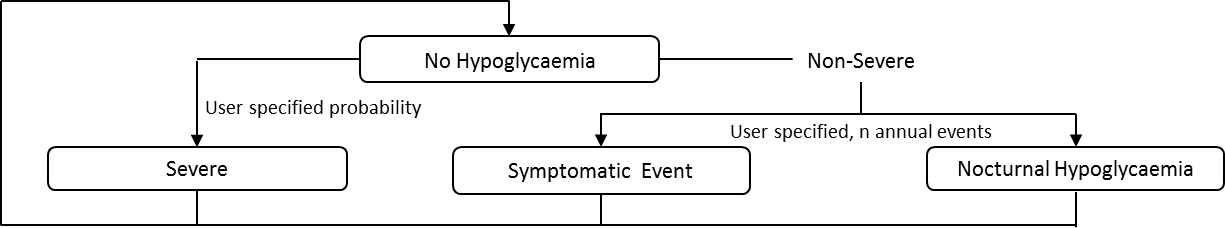


Figure 15: Modelling of hypoglycaemia

## All-cause mortality

All-cause mortality (ACM) is modelled using gender-specific UK life tables.^17^ The annual probability of ACM is converted to a 6-monthly probability and during each cycle the likelihood of such an event is assessed. All patients incur the risk of ACM. Due to the relatively low contribution of T1DM to the incidence of ACM, life tables have not been modified to account for potential double-counting of mortality events. The potential double counting is unlikely to significantly bias cost-effectiveness results.

## Summary of model equations and rates

Table 15 shows a summary of equations and rates utilised within the model, including a brief description and a list of sources.

Table 16: Summary of equations and rates used within the model

| **Risk equation/rate** | **Description** | **Source** |
| --- | --- | --- |
| Eq 1 | No retinopathy to background retinopathy – Equation fitted to DCCT/EDIC data | DCCT/EDIC^2^ |
| Eq 2 | Background retinopathy to proliferative retinopathy – Equation fitted to DCCT/EDIC data | DCCT/EDIC^2^ |
| Eq 3 | Proliferative retinopathy to severe vision loss – Equation fitted to DCCT/EDIC data | DCCT/EDIC^2^ |
| Eq 4 | No macular edema to macular edema – Equation fitted to DCCT/EDIC data | DCCT/EDIC^2^ |
| Eq 5 | Macular edema to severe vision loss – Equation fitted to DCCT/EDIC data | DCCT/EDIC^2^ |
| Eq 6 | No nephropathy to micro-albuminuria – Equation fitted to DCCT/EDIC data | De Boer *et al.*^3^ |
| Eq 7 | Micro-albuminuria to no nephropathy – Equation fitted to DCCT/EDIC data | De Boer *et al.*^3^ |
| Eq 8 | Micro-albuminuria to macro-albuminuria – Equation fitted to DCCT/EDIC data | De Boer *et al.*^3^ |
| Eq 9 | Micro-albuminuria to macro-albuminuria with impaired GFR – Equation fitted to DCCT/EDIC data | De Boer *et al.*^3^ |
| Eq 10 | Macro-albuminuria/macro-albuminuria with impaired GFR to ESRD – Equation fitted to DCCT/EDIC data | De Boer *et al.*^3^ |
| Eq 11 | No neuropathy to diabetic peripheral neuropathy – Equation fitted to DCCT/EDIC data | Martin *et al.^1^* |
| Eq 12 | No CVD to CVD and CVD to subsequent CVD – Published equation fitted to Swedish NDR data utilised | Cederholm *et al.^6^* |
| Rate 1 | Dialysis to transplant – Rate based upon 5 year treatment switching data reported by the UK renal registry | UK Renal Registry^8^ |
| Rate 2 | Transplant to dialysis – Rate based upon contemporary study reporting 5 year rates of graft and overall transplant survival | Taber *et al.^9^* |
| Rate 3 | Dialysis to death – Rate based upon the annual dialysis survival rate in patients with diabetes as their primary renal diagnosis, as reported in the UK renal registry | UK Renal Registry^14^ |
| Rate 4 | Transplant to death – Rate based upon contemporary study reporting 5 year rates of graft and overall transplant survival | Taber *et al.^9^* |
| Rate 5 | Diabetic peripheral neuropathy to uncomplicated foot ulcer – Rate based on comprehensive published Markov model describing ulcer and amputation disease states | Tennvall and Apelqvist^5^ |
| Rate 6 | Diabetic peripheral neuropathy to deep foot infection – Rate based on comprehensive published Markov model describing ulcer and amputation disease states | Tennvall and Apelqvist^5^ |
| Rate 7 | Diabetic peripheral neuropathy to foot ulcer and critical ischaemia – Rate based on comprehensive published Markov model describing ulcer and amputation disease states | Tennvall and Apelqvist^5^ |
| Rate 8 | Uncomplicated foot ulcer to diabetic peripheral neuropathy – Rate based on comprehensive published Markov model describing ulcer and amputation disease states | Tennvall and Apelqvist^5^ |
| Rate 9 | Deep foot infection to diabetic peripheral neuropathy – Rate based on comprehensive published Markov model describing ulcer and amputation disease states | Tennvall and Apelqvist^5^ |
| Rate 10 | Deep foot infection to minor amputation – Rate based on comprehensive published Markov model describing ulcer and amputation disease states | Tennvall and Apelqvist^5^ |
| Rate 11 | Deep foot infection to major amputation – Rate based on comprehensive published Markov model describing ulcer and amputation disease states | Tennvall and Apelqvist^5^ |
| Rate 12 | Foot ulcer and critical ischaemia to diabetic peripheral neuropathy – Rate based on comprehensive published Markov model describing ulcer and amputation disease states | Tennvall and Apelqvist^5^ |
| Rate 13 | Foot ulcer and critical ischaemia to minor amputation – Rate based on comprehensive published Markov model describing ulcer and amputation disease states | Tennvall and Apelqvist^5^ |
| Rate 14 | Foot ulcer and critical ischaemia to major amputation – Rate based on comprehensive published Markov model describing ulcer and amputation disease states | Tennvall and Apelqvist^5^ |
| Rate 15 | Diabetic peripheral neuropathy to death – Rate based on comprehensive published Markov model describing ulcer and amputation disease states | Tennvall and Apelqvist^5^ |
| Rate 16 | Uncomplicated foot ulcer to death – Rate based on comprehensive published Markov model describing ulcer and amputation disease states | Tennvall and Apelqvist^5^ |
| Rate 17 | Deep foot infection to death – Rate based on comprehensive published Markov model describing ulcer and amputation disease states | Tennvall and Apelqvist^5^ |
| Rate 18 | Foot ulcer and critical ischaemia – Rate based on comprehensive published Markov model describing ulcer and amputation disease states | Tennvall and Apelqvist^5^ |
| Rate 19 | Minor amputation to death – Rate based on comprehensive published Markov model describing ulcer and amputation disease states | Tennvall and Apelqvist^5^ |
| Rate 20 | Major amputation to death – Rate based on comprehensive published Markov model describing ulcer and amputation disease states | Tennvall and Apelqvist^5^ |
| Rate 21 | No ketoacidosis to ketoacidosis – Rate of 1.585 events per 100,000 patient years | Wang *et al*.^7^ |
| Rate 22 | CVD/subsequent CVD to death – Rate of 39.19% applied to all incident events, based on a contemporary study | Rawshani *et al.^15^* |

BDR: background diabetic retinopathy; CON: conventional therapy; DD: diabetes duration; DBP: diastolic blood pressure; DPN: diabetic peripheral neuropathy; ESRD: end-stage renal disease; GFR: glomerular filtration rate; Hba1c: haemoglobin A 1c; MAP: mean arterial pressure; μAU: Micro-albuminuria; MAU: Macro-albuminuria; PDR: proliferative diabetic retinopathy; SBP: systolic blood pressure; SVL: severe vision loss.

# References

1. Martin CL, Albers JW, Pop-Busui R. Neuropathy and related findings in the diabetes control and complications trial/epidemiology of diabetes interventions and complications study. Diabetes Care. 2014;37(1):31-8.

2. Diabetes Control and Complications Trial/Epidemiology of Diabetes Interventions and Complications Research G. Effect of intensive diabetes therapy on the progression of diabetic retinopathy in patients with type 1 diabetes: 18 years of follow-up in the DCCT/EDIC. Diabetes. 2015;64(2):631-42.

3. de Boer IH. Kidney disease and related findings in the diabetes control and complications trial/epidemiology of diabetes interventions and complications study. Diabetes care. 2014;37(1):24-30.

4. de Boer IH, Rue TC, Cleary PA, Lachin JM, Molitch ME, Steffes MW, et al. Long-term renal outcomes of patients with type 1 diabetes mellitus and microalbuminuria: an analysis of the Diabetes Control and Complications Trial/Epidemiology of Diabetes Interventions and Complications cohort. Archives of internal medicine. 2011;171(5):412-20.

5. Tennvall GR, Apelqvist J. Prevention of diabetes-related foot ulcers and amputations: a cost-utility analysis based on Markov model simulations. Diabetologia. 2001;44(11):2077-87.

6. Cederholm J, Eeg‐Olofsson K, Eliasson B, Zethelius B, Gudbjörnsdottir S. A new model for 5‐year risk of cardiovascular disease in Type 1 diabetes; from the Swedish National Diabetes Register (NDR). Diabetic Medicine. 2011;28(10):1213-20.

7. Wang ZH, Kihl‐Selstam E, Eriksson JW. Ketoacidosis occurs in both Type 1 and Type 2 diabetes—a population‐based study from Northern Sweden. Diabetic Medicine. 2008;25(7):867-70.

8. UK Renal Registry. UK Renal Registry 17th Annual Report: Chapter 1 UK Renal Replacement Therapy Incidence in 2013: National and Centre-specific Analyses. 2014. Available from: <https://www.renalreg.org/wp-content/uploads/2014/12/01-Chap-01.pdf>. [Accessed 18 September 2015].

9. Taber DJ, Meadows HB, Pilch NA, Chavin KD, Baliga PK, Egede LE. Pre‐existing diabetes significantly increases the risk of graft failure and mortality following renal transplantation. Clinical transplantation. 2013;27(2):274-82.

10. Engauge. Available from: <http://digitizer.sourceforge.net/>. [Accessed 01 August 2015].

11. Diabetes Control and Complications Trial Research Group. The effect of intensive treatment of diabetes on the development and progression of long-term complications in insulin dependent diabetes mellitus. The New England journal of medicine. 1993;329(14):977-86.

12. National Institue for Health and Care Excellence. NICE guidelines [NG17]. Type 1 diabetes in adults: diagnosis and management. 2015. Available from: <https://www.nice.org.uk/guidance/ng17>. [Accessed 01 September 2015].

13. Javitt JC, Aiello LP, Chiang Y, Ferris FL, 3rd, Canner JK, Greenfield S. Preventive eye care in people with diabetes is cost-saving to the federal government. Implications for health-care reform. Diabetes Care. 1994;17(8):909-17.

14. UK Renal Registry. UK Renal Registry 17th Annual Report: Chapter 5 Survival and Cause of Death in UK Adult Patients on Renal Replacement Therapy in 2013: National and Centre-specific Analyses. 2014. Available from: <https://www.renalreg.org/wp-content/uploads/2014/12/05-Chap-05.pdf>. [Accessed 18 September 2015].

15. Rawshani A, Svensson A-M, Rosengren A, Eliasson B, Gudbjörnsdottir S. Impact of Socioeconomic Status on Cardiovascular Disease and Mortality in 24,947 Individuals With Type 1 Diabetes. Diabetes care. 2015:dc150145.

16. Nationella Diabetesregistret. The Swedish National Diabetes Register (NDR) – Centre of Registers, Region Västra Götaland. 2015. Available from: <https://www.ndr.nu/>. [Accessed 4 December 2015].

17. UK office for National Statistics. England and Wales life tables: 1980-82 to 2012-2014. Available from: [http://www.ons.gov.uk/ons/taxonomy/index.html?nscl=Life+Tables - tab-data-tables](http://www.ons.gov.uk/ons/taxonomy/index.html?nscl=Life+Tables#tab-data-tables). [Accessed 16 October 2015].
